# Protocol for a single-arm, prospective mixed-methods feasibility study of a culturally responsive maternal health promotion intervention

**DOI:** 10.3389/fgwh.2026.1726319

**Published:** 2026-04-13

**Authors:** Jennifer R. Budman, Helen Bourke-taylor

**Affiliations:** 1 Hadassah Medical School, Faculty of Medicine, Hebrew University of Jerusalem, Jerusalem, Israel; 2 Department of Occupational Therapy, Monash University, Melbourne, VIC, Australia

**Keywords:** ADHD, cultural competency, group intervention, health promotion, maternal stress

## Abstract

**Background:**

Maternal mental health is a critical determinant of women's health and family functioning, yet mothers of children with Attention-Deficit/Hyperactivity Disorder (ADHD) experience elevated stress, stigma, and barriers to health-promoting behaviors. These challenges are intensified in culturally conservative communities, where gender-role expectations and limited access to culturally appropriate services restrict engagement with mainstream health care.

**Objective:**

This protocol outlines the implementation, and evaluation of a culturally responsive maternal health promotion intervention for Ultra-Orthodox Jewish (UOJ) mothers of children with ADHD. Earlier phases of the Intervention Mapping process, including needs assessment and intervention development, were completed prior to this protocol. The present study focuses on the final two steps of Intervention Mapping, implementation and evaluation, to support that the program is deliverable, acceptable, and sustainable in real-world practice.

**Methods:**

This protocol outlines a prospective, single-arm, mixed-methods feasibility study without a control group, to be conducted with UOJ mothers in telehealth-based groups. The six-session program integrates psychoeducation, peer discussion, and action planning, supported by culturally adapted materials and a moderated WhatsApp group. Implementation and acceptability will be evaluated through recruitment, retention, attendance, and structured feedback surveys, supplemented by qualitative focus groups. Intervention outcomes will include maternal stress, ADHD-related knowledge and beliefs, stigma, and engagement in health-promoting activities, assessed using validated instruments. Quantitative data will be analyzed using descriptive and repeated-measures statistics, and qualitative data will undergo thematic analysis.

**Conclusion:**

By integrating systematic planning with cultural tailoring, this protocol aims to contribute a model for developing and evaluating women's health interventions in underserved, culturally conservative populations. The study seeks to advance maternal health equity by demonstrating how culturally responsive approaches may reduce barriers, foster engagement, and promote sustainable health-promoting practices.

**Trial status:**

Recruitment commenced in November 2024 and remains ongoing at the time of manuscript submission. No interim analyses or outcome data analyses have been conducted at the time of manuscript submission.

## 1 Introduction

Maternal mental health and well-being are fundamental to women's health and family functioning (1). Mothers of children with Attention Deficit Hyperactivity Disorder (ADHD), a common neurodevelopmental disorder affecting 5%–7% of children worldwide, carry a disproportionate burden of stress, caregiving responsibility, and reduced quality of life compared to mothers of children without ADHD (2, 3). Chronic exposure to such stress is associated with heightened risks of depression, anxiety, burnout, and adverse physical health outcomes (3). These findings highlight the need for interventions that explicitly target maternal well-being in families affected by ADHD (2–4).

Women in culturally conservative and traditionally religious communities often face additional inequities that compound caregiving challenges (5). They experience barriers such as stigma surrounding mental health, limited access to culturally appropriate services, and strong gender-role expectations (6–8). These factors often increase isolation, discourage help-seeking, and limit engagement in health-promoting behaviors (9). One such example of a culturally conservative population is the UOJ community. The UOJ community is a rapidly growing religious minority characterized by strong adherence to traditional Jewish law, rabbinical authority, and cultural separation from secular society. Families are typically large, and gender roles are clearly delineated, with women primarily responsible for household management and childrearing, while also frequently serving as the main income earners to support their partners extended religious studies (10). Community values emphasize modesty, conformity, and reliance on internal support systems, while engagement with mainstream health and social services is often limited or viewed with caution (7, 11, 12). Mental health concerns, including ADHD, are often stigmatized, and formal supports underutilized (7). These cultural and structural factors have been shown to intensify mothers’ caregiving burden (13) and underscore the importance of culturally responsive health promotion approaches (4, 13). However, similar dynamics have been described in other culturally conservative or minority populations worldwide, including Muslim (14), Hispanic (15), and Indigenous communities (16), where stigma surrounding mental health, restrictive gender roles, and limited access to culturally responsive care constrain women's participation in health-promoting behaviors and help-seeking. Addressing these barriers is therefore not only critical within the UOJ community but also represents an essential priority for women's health promotion and health equity globally in comparable populations (1, 5, 14–16).

To meet these challenges, systematic approaches such as the Intervention Mapping (IM) framework provide a structured, theory-driven process for developing interventions that are evidence-based, culturally responsive, and sustainable (17). Intervention Mapping integrates theoretical foundations, empirical evidence, and stakeholder input to guide the systematic development of health promotion programs. The framework involves six iterative steps: (1) conducting a needs assessment to identify key health issues and their behavioral and contextual determinants; (2) formulating specific and measurable program objectives; (3) selecting theory-based methods and practical strategies to address these determinants; (4) developing intervention components and materials that are tailored to the target population's cultural and social context; (5) planning for implementation and sustainability; and (6) creating an evaluation plan to assess feasibility, effectiveness, and process outcomes. By emphasizing both rigor and contextual sensitivity, the IM framework offers a useful guide for adapting health promotion interventions to the needs of diverse and underserved populations (17).

Earlier intervention development work completed and reported IM steps 1–4, including needs assessment, specification of program objectives, selection of theory-based methods, and development of culturally responsive intervention materials (4). Building on this foundation, the present manuscript focuses specifically on Steps 5 and 6 of the IM framework, which address implementation planning and evaluation design. Reporting distinct phases of the IM process in separate publications is consistent with its staged and iterative structure, particularly when intervention development, implementation planning, and evaluation occur over extended timelines (17). These final steps are important for ensuring that the intervention is feasible in real-world settings and appropriately aligned with the cultural context of the target population (17). Thus, the aim of this paper is to present the study protocol for the implementation and evaluation of a culturally responsive maternal health promotion intervention for UOJ mothers of children with ADHD within a culturally conservative and traditionally religious community. By detailing the implementation strategies and evaluation framework, guided by IM steps 5 and 6, this protocol provides a model for integrating cultural responsiveness into maternal health intervention planning in culturally conservative communities and contributes to broader efforts to advance health equity (5, 7, 8).

The UOJ community was selected as the focus of this study as the first author has an established history of collaboration and research within this community, which has helped to build trust, cultural understanding, and ethical engagement with participants (4, 9, 13, 18). In addition, the social and structural characteristics of the UOJ community reflect challenges shared by other culturally conservative and traditionally religious communities worldwide. For this reason, insights from this setting may offer transferable lessons for developing culturally responsive maternal health promotion programs in similar contexts (6, 7, 9, 13–16).

## 2 Methods and analysis

### 2.1 Ethical approval and registration

Ethical approval for this study was obtained from the University's Institutional Review Board (IRB #30012024). The study was prospectively registered at ClinicalTrials.gov (NCT06703242). All participants will provide informed consent before enrollment.

### 2.2 Study design and setting

This study describes the protocol of a prospective, single-arm, mixed-methods feasibility study without a control group, to be conducted with UOJ mothers in telehealth-based groups. The study integrates quantitative and qualitative methods to assess program implementation, acceptability, and potential effects on maternal health outcomes (18–20). The intervention will be delivered remotely via Zoom in one country Israel.

### 2.3 Recruitment and consent

Recruitment will take place through culturally appropriate channels, including accepted social media platforms used within the UOJ community, child development centers situated in UOJ neighborhoods, and peer referral. Recruitment materials will emphasize the program's health promotion focus, confidentiality of participation, and alignment with cultural values, to foster trust and support mothers' sense of safety (5, 7, 8, 13, 18). Interested mothers will be screened via telephone for eligibility and invited to provide written informed consent electronically by the first author.

Given the close-knit nature of the UOJ community and the first author's involvement in screening and consent procedures, specific measures will be taken to minimize potential coercion. Recruitment materials and consent procedures will explicitly emphasize the voluntary nature of participation, and participants will be informed that declining or withdrawing will not affect access to services or future support. Standardized consent scripts will be used to ensure consistency (16, 21), and the study has received IRB approval to safeguard ethical conduct.

### 2.4 Sample size

A formal power calculation was not undertaken, as the primary aim of this protocol is to describe and evaluate implementation rather than to test efficacy (22–24). The planned recruitment target is 40–48 participants, organized into eight groups of 6–8 mothers each. This group size was selected to balance diversity of perspectives with manageable group dynamics that support peer learning and meaningful interaction (25). Although not powered to detect definitive treatment effects, this sample size supports use of descriptive statistics, repeated measures analyses, and exploratory subgroup comparisons related to cultural dynamics (e.g., mothers of sons vs. daughters).

### 2.5 Intervention procedures

The intervention consists of six weekly, 90-minute sessions delivered via telehealth (Zoom) to enhance accessibility for working mothers. Each session follows a structured format: a culturally adapted presentation introducing the weekly topic, active learning and peer discussion, and action planning to encourage application of skills in daily life. Content is organized into three thematic modules: (1) knowledge of ADHD and stigma reduction (four sessions), (2) managing ADHD in school and strengthening advocacy skills (one session), and (3) promoting maternal health behaviors (one session). Additionally, weekly digital handouts and action-planning sheets will be provided electronically to reinforce session content. A moderated WhatsApp group will be provided to support ongoing peer support, reflection, and shared problem-solving between sessions (26). One session will include an educator to facilitate dialogue about parent–school collaboration. The program will be delivered by an occupational therapist with expertise in maternal health and cultural adaptation (4).

Although only one session directly targets maternal health behaviors, the intervention conceptualizes maternal health promotion as encompassing psychosocial determinants of well-being. ADHD-related knowledge, stigma reduction, and school advocacy skills are addressed as upstream factors that influence maternal stress, caregiving burden, self-efficacy, and engagement in health-promoting behaviors (2–4, 7, 13). Prior research has demonstrated that caregiving stress, stigma, and limited access to culturally responsive supports contribute significantly to maternal mental and physical health risks in families affected by ADHD (3, 7, 13). By targeting these interconnected domains, the program aims to support maternal health both directly and indirectly within the broader caregiving context.

Additionally, cultural considerations will be integrated by using language and visuals that are familiar to the community, with sessions delivered in the group's primary language, or through bilingual delivery (English) as needed (9). Potential barriers, such as lack of internet access or stigma around group participation, will be addressed through measures like offering internet sticks and leveraging stakeholder commitment, particularly educators, to encourage participation.

The structure and delivery of the program were intentionally designed to align with the cultural values, family structures, and practical realities of UOJ mothers. The use of short, focused modules respects participants' limited time and high caregiving load (8–13). Incorporating group discussion and peer exchange reflects mothers' need for collective learning and mutual support (4), whereas structured action-planning tasks promote concrete functional skill use consistent with behavioral principles (27). The inclusion of digital materials and a moderated WhatsApp group extends learning beyond formal sessions, providing continuity in a format that is culturally responsive and used within the community (4).

Participants will be able to access additional care during the intervention. Information about this care, such as medication or psychotherapy, will be collected during post-intervention and follow-up surveys. These surveys will include questions like: “Had you used any mental health or parenting resources during the intervention?”, “What types of resources did you use?”, and “How effective were these resources in addressing your needs?” (See supplemental file 3, Focus group interview guide)

### 2.6 Implementation strategies

Implementation strategies were developed in line with Step 5 of the Intervention Mapping framework (17), focusing on adoption, accessibility, and sustainability within the UOJ community (28). Evening scheduling and telehealth delivery reflect the reality that many UOJ mothers are primary income earners with limited daytime availability (13, 29). Cultural and linguistic adaptations, including familiar language, visuals, and optional bilingual delivery, aim to enhance program relevance and reduce barriers related to stigma or perceived inappropriateness of external interventions (7, 8).

Facilitator preparation will include training in cultural competence and group processes, with delivery by an occupational therapist experienced in maternal health and community engagement (28–30). To strengthen community buy-in, one session will feature an educator to bridge parent–school collaboration, and partnerships with schools and community organizations will be explored to support sustainability (9). Within the UOJ community, schools play a central role not only in children's education but also in shaping and upholding communal norms and values. A child's school performance often reflects the family's reputation and religious commitment, making school engagement a matter of both practical and social importance (4, 8). Involving educators in the intervention is therefore intended to enhance the program's cultural acceptability and relevance, while positioning maternal participation within an institution that is trusted and respected in the community. Additionally, practical supports such as text message reminders and, if needed, access to internet devices will help minimize barriers to participation (11, 12, 28). These measures are particularly important given that some UOJ households have limited or filtered internet access for religious reasons, which may restrict participation in online programs (11, 12). By offering alternative ways to connect and providing gentle technological support, the program aims to ensure inclusivity while respecting community norms and values. These strategies reflect the integration of both cultural tailoring and behavior change principles to support long-term engagement (26–29).

### 2.7 Evaluation indicators and outcome measures

Evaluation was guided by Step 6 of the Intervention Mapping framework (17), with a dual focus on implementation assessment and maternal outcomes.

#### 2.7.1 Implementation and acceptability

Will be assessed through recruitment and retention rates, session attendance, and completion of action-planning tasks, as well as structured feedback surveys after each session (18, 20, 31). Predefined feasibility benchmarks have been established to guide interpretation. Recruitment will be considered feasible if at least 75% of the planned sample is enrolled within the recruitment period. Retention will be considered acceptable if at least 80% of enrolled participants complete the six-session program and post-intervention assessments. Attendance feasibility will be defined as at least 75% of participants attending four or more sessions. Acceptability will be considered high if mean session feedback scores are ≥3 (“moderately”) on relevance, effectiveness, and cultural appropriateness scales. Establishing predefined progression criteria is consistent with feasibility study guidance (18, 20). These surveys use a four-point Likert scale to capture perceived relevance, effectiveness, and cultural appropriateness (31). At program completion, qualitative focus groups will provide additional feedback on content acceptability, cultural fit, and perceived usefulness (18, 20). These benchmarks will inform decisions regarding refinement of recruitment strategies or progression to a future controlled trial.

#### 2.7.2 Maternal outcomes

Will be evaluated with validated instruments reflecting the intervention's objectives. Maternal stress will be measured using the Parental Stress Items (PSI) (32), as stress is a primary determinant of maternal well-being and a well-documented burden among mothers of children with ADHD (2, 5), including such mothers in the UOJ community (9). ADHD-related knowledge and beliefs will be assessed with the Parental ADHD Knowledge and Beliefs Questionnaire (33), given that knowledge gaps and misconceptions were expressed by stakeholders in the UOJ community to be determinants that impact effective management and advocacy (8, 9). Stigma will be measured with the ADHD Stigma Questionnaire (34), recognizing stigma as a major barrier to care in the UOJ community and similar populations worldwide (8, 14–16). Finally, maternal health behaviors will be measured using the Health Promoting Activities Scale (HPAS) (1), reflecting the program's emphasis on supporting women's engagement in self-care practices that build resilience and reduce stress (1). Together, these instruments attempt to provide a multidimensional picture of maternal mental health, health literacy, and engagement in health-promoting practices, while remaining sensitive to the cultural and social determinants that shape women's health experiences (17).

The selected instruments have demonstrated reliability and validity in general populations and have been used in ADHD and maternal health research. All instruments have previously translated Hebrew versions. However, these instruments have not been formally validated within the UOJ community. To address potential cultural nuances in interpretation, qualitative focus groups will explore the contextual relevance and clarity of questionnaire items. Findings will inform future work on cultural adaptation and validation within this population.

### 2.8 Data collection and timeline

Data collection will occur across four stages. During enrollment (weeks 1–8), participants will complete eligibility screening, provide consent, and complete baseline questionnaires. The intervention will run between weeks 10–21, with participants enrolled in staggered cohorts. Post-intervention data, including follow-up questionnaires and focus groups, will be collected between weeks 16–23. Due to staggered cohort initiation, post-intervention assessments for earlier cohorts may overlap with ongoing sessions for later cohorts; however, within each cohort, post-intervention data will be collected only after completion of all six sessions. A final reassessment will be conducted between weeks 28–35, approximately three months after program completion, to evaluate sustainability of outcomes (see Table 1).

**Table 1:** SPIRIT schedule of enrolment, interventions, and assessments.

Study period	Enrollment,	Allocation	Intervention	Post intervention	Follow up
Timepoint	Weeks 1-8	Weeks 8-9	Weeks 10-21	Weeks 16-23	Weeks 28-35
Informed consent	X				
Eligibility screener	X				
Allocation		X			
Preliminary evaluations			X		
Intervention			X		
Quantitative outcome evaluations Focus groups				X	X X

Participants will be enrolled in staggered cohorts. Post-intervention assessments occur only after completion of the six-session program for each cohort; overlapping weeks reflect staggered cohort scheduling.

### 2.9 Data analysis

All recruited participants with baseline data will be included in the primary analyses, consistent with an intention-to-treat approach. Anonymized quantitative data collected via QuestionPro will be transferred to the IBM Statistical Package for the Social Sciences (SPSS version 27, Chicago, IL, USA). Descriptive statistics will summarize categorical (frequencies and percentages) and continuous variables (means and standard deviations). For continuous outcomes, repeated-measures analyses of variance (ANOVA) will be conducted to examine changes across time points (baseline, post-intervention, and follow-up). Effect sizes will be reported using partial eta squared. Assumptions of normality and sphericity will be assessed; where the sphericity assumption is violated, Greenhouse–Geisser corrections will be applied. If data distributions substantially deviate from normality, appropriate non-parametric alternatives will be considered (35). Missing data will be examined for extent and pattern. If missingness is minimal (<5%) and determined to be missing at random, appropriate imputation methods will be applied. Sensitivity analyses will be conducted as needed to assess potential impact (35). Qualitative data from focus groups will be transcribed verbatim by a blinded master's student and analyzed thematically by the authors, guided by the COREQ framework (36). Coding will follow an inductive approach, with credibility enhanced through triangulation, member checking, and peer debriefing among the research team.

### 2.10. Rigor, oversight, and data protection

To enhance methodological rigor, an advisory panel comprising maternal health and cultural/religious experts will provide oversight throughout implementation and offer periodic feedback (19). Strategies to strengthen credibility include triangulation of quantitative and qualitative data, member checking, and peer debriefing (29, 31). Participant data will be anonymized and stored in encrypted electronic files accessible only to the research team. Findings will be disseminated through peer-reviewed publications and community presentations.

Confidentiality procedures will be reviewed with participants at the outset of the program to promote respectful group engagement. While confidentiality cannot be fully guaranteed in group-based interventions, clear expectations will be established to minimize risk, consistent with ethical recommendations (37). Participants experiencing distress during sessions will be supported by the facilitator and, when appropriate, provided with referrals to external mental health services.

## 3 Discussion

This study protocol describes the design and planned implementation and evaluation of a culturally responsive health promotion program for Ultra-Orthodox Jewish (UOJ) mothers of children with ADHD. The program seeks to address maternal stress, stigma, and ADHD-related knowledge while promoting engagement in health-promoting behaviors. By presenting the protocol transparently, this article responds to calls for the development of evidence-based, equity-oriented interventions that strengthen maternal mental health in underserved populations (21, 29).

The intervention was developed with explicit attention to the cultural realities of the UOJ community. Mothers in this setting often serve as primary income earners while simultaneously carrying full responsibility for childrearing and household management (6, 7). This dual role contributes to elevated stress and limited opportunities for self-care (4, 9, 13). Accordingly, evening telehealth delivery was chosen to align with participants' schedules, ensuring accessibility without compromising daily responsibilities (26–29). The moderated WhatsApp group was added to extend peer support beyond structured sessions in a familiar and acceptable format. Because many mothers in this community report feeling isolated and without a supportive peer network (4), this feature was intended to foster connectedness (27, 30). Grouping mothers by whether they were raising sons or daughters further reflects gendered expectations within the community, acknowledging distinct caregiving and educational challenges (8). Such adaptations are not peripheral; they are essential to ensuring that the intervention resonates with participants and reduces barriers to participation (8, 9).

A further strength of this protocol is the application of the IM framework to guide implementation planning and evaluation strategies. While earlier phases of intervention development (Steps 1–4) were reported previously (4), the present manuscript operationalizes Steps 5 and 6, thereby ensuring that implementation and evaluation are grounded in a structured and theory-informed process. While cultural tailoring shaped content and delivery, IM provided the scaffolding to translate community needs into clear objectives, implementation strategies, and outcomes (17). This framework enhances methodological rigor and transparency while supporting the replicability of the program in other culturally specific contexts.

The role of occupational therapists as facilitators also strengthens the intervention. Occupational therapy emphasizes participation, caregiver roles, and health-promoting behaviors, offering a perspective that bridges evidence-based practice with community-specific adaptation and function in life roles (38). Involving culturally knowledgeable professionals supports trust and acceptability (8), both of which are critical in communities where stigma and skepticism toward external interventions are common (9).

Although tailored to the UOJ community, the principles underpinning this program may be transferable to other religious or minority populations facing similar barriers. Many groups worldwide contend with stigma, rigid gender roles, and limited access to culturally responsive care (14–16, 29). Documenting both the cultural tailoring and systematic development process may provide a roadmap for adaptation and application in diverse contexts (17).

Beyond its relevance to the UOJ community, this study contributes to the broader field of implementation science and women's health equity. Documenting not only the intervention design but also the planned implementation strategies and evaluation framework offers a model for how culturally responsive interventions may be systematically developed, implemented and assessed (17). Many maternal health programs struggle to translate into practice because they are less sensitive to the cultural, social, and structural contexts in which women live (4, 10, 29). By applying Intervention Mapping to support both cultural tailoring and methodological rigor, this protocol demonstrates how equity-oriented approaches may bridge the gap between program development and sustainable delivery (28). In this way, the study attempts to advance knowledge on adapting health promotion interventions for underserved groups and contributes to global efforts to reduce disparities in women's health.

### 3.1 Potential limitations

Several limitations should be acknowledged. As a single-arm feasibility protocol without a control group, the study is not designed to establish causal effects; any observed changes in outcomes will be interpreted cautiously as preliminary. The planned sample size is appropriate for initial protocol evaluation but will not yield generalizable findings; future randomized controlled trials with larger samples will be needed to evaluate effectiveness rigorously. Although the selected outcome measures are psychometrically validated, they may not fully capture the nuanced experiences of UOJ mothers, and their cultural validity will need to be explored during this study. Additionally, the first author's involvement in recruitment, facilitation, and aspects of data collection may introduce potential bias. While standardized procedures and advisory oversight are in place, role concentration may influence participant responses or interpretation of findings. Technology-related challenges, including inconsistent internet access and varying digital literacy, may also disrupt program delivery, despite mitigation strategies such as provision of internet devices and technical support. Given the close-knit nature of the UOJ community, social desirability bias may also affect self-reported outcomes, particularly regarding stigma and health behaviors. Efforts to mitigate this include emphasizing confidentiality and voluntary participation; however, this remains an inherent consideration in community-based research.

### 3.2 Risk mitigation

Several strategies were built into the protocol to minimize risks. Confidentiality will be safeguarded through clear group agreements, with additional steps such as pre-intervention confidentiality contracts considered for future studies (4, 37). Participants unable to uphold these standards will continue to receive psychoeducational materials and referrals to external resources. The occupational therapist leading the program will monitor for signs of distress and provide tailored referrals when necessary. These measures align with ethical recommendations for group-based interventions and are intended to ensure participant safety (37).

### 3.3 Future directions

This protocol represents an initial step toward implementing and evaluating a culturally responsive maternal health promotion program for UOJ mothers of children with ADHD. It provides groundwork for larger trials examining outcomes such as stress reduction, improved ADHD knowledge, reduced stigma, and enhanced maternal health behaviors. Future studies should also explore long-term effects. Embedding the program in schools, healthcare systems, and community organizations may also strengthen sustainability and community ownership. Addressing stigma and supporting maternal advocacy within the UOJ community may further enhance impact by promoting acceptance and engagement. More broadly, this work illustrates how systematic planning, and cultural tailoring can be integrated to guide the development of interventions in underserved populations, with potential transferability beyond the immediate context.

## 4 Conclusion

This protocol outlines the design, implementation, and evaluation of a culturally responsive health promotion intervention for UOJ mothers of children with ADHD. By addressing maternal stress, reducing stigma, strengthening ADHD-related knowledge, and promoting health-supporting behaviors, the program aims to empower mothers while acknowledging the realities of their cultural and social context. The systematic application of the IM framework ensures that adaptations are purposeful, transparent, and transferable. Although developed for the UOJ community, the processes outlined (i.e., cultural tailoring, systematic planning, and integration of equity-oriented strategies) may offer a model for designing maternal health interventions in other culturally conservative and traditionally religious populations. In this way, this study seeks to contribute not only to advancing maternal mental health and caregiving support but also to broader public health efforts toward gender equity and reducing disparities in access to care.
